# Detection of *Mycoplasma haemocanis*, *Mycoplasma haematoparvum*, *Mycoplasma suis* and other vector-borne pathogens in dogs from Córdoba and Santa Fé, Argentina

**DOI:** 10.1186/s13071-016-1920-8

**Published:** 2016-12-15

**Authors:** Patricia E. Mascarelli, Gustavo P. Tartara, Norma B. Pereyra, Ricardo G. Maggi

**Affiliations:** 1 Intracellular Pathogens Research Laboratory, Department of Clinical Sciences, College of Veterinary Medicine, North Carolina State University, 1060 Williams Moore Dr., Raleigh, NC 27607 USA; 2 Cátedra de Microbiología, Facultad de Ciencias Veterinarias, Universidad Nacional de Rosario, Bv. Spangemberg and Bv. Colón, 2170 Casilda, Santa Fé Argentina

**Keywords:** Dogs, *Babesia vogeli*, *Anaplasma phagocytophilum*, *Anaplasma platys*, *Ehrlichia canis*, *Mycoplasma haemocanis*, *Mycoplasma haematoparvum*, *Mycoplasma suis*, *Bartonella* spp., Argentina

## Abstract

**Background:**

In Argentina, only very few reports are available for canine tick-borne diseases where most are related to parasitic diseases. The objective of this survey was to investigate the prevalence of tick-borne pathogens in 70 dogs from Santa Fé and Córdoba, Argentina.

**Methods:**

Microscopic blood smear examination as well as polymerase chain reaction (PCR) amplification using species-specific markers of *Anaplasma*, *Babesia*, *Bartonella*, *Borrelia*, *Ehrlichia*, *Francisella*, *Mycoplasma* (hemotropic group) and *Rickettsia*, followed by DNA sequencing were used to establish the prevalence of each infecting pathogen.

**Results:**

Blood smear analysis showed 81% (57/70) prevalence of structures morphologically compatible with hemotropic mycoplasmas. No structures resembling either piroplasms or *Anaplasma/Ehrlichia* were detected. Hemotropic mycoplasma species (*Mycoplasma haematoparvum*, *Mycoplasma haemocanis* and *Mycoplasma suis*) were the most prevalent pathogens detected with an overall prevalence of 77.1%. *Anaplasma platys* was detected and identified in 11 of the 70 dogs (15.7%), meanwhile two *Bartonella* spp. (*B. clarridgeiae* and an uncharacterized *Bartonella* sp.) and *Babesia vogeli* were detected at 3 and 7% prevalence, respectively.

**Conclusions:**

The work presented here describes a high molecular prevalence for hemotropic mycoplasma species in each of the five locations selected. Three *Mycoplasma* spp., including *Mycoplasma suis*, reported for the first time in dogs have been identified by DNA amplification and sequencing. This study highlights the risk that these bacterial pathogens represent for companion animals and, due to their potential zoonotic nature, also for people.

## Background

The detection of canine vector-borne pathogens represents a constant challenge for veterinarians and researchers. The wide array of pathogenic organisms (protozoa, bacteria and viruses), their diverse biologic behavior and distribution, and the wide spectrum of clinical signs contribute to the difficulties associated with the diagnosis of canine vector-borne diseases (CVBD).

Only few reports are available for CVBD in Argentina and, as in many countries in Latin American, most are related to parasitic diseases such as babesiosis [1, 2], hepatozoonosis [2, 3], dirofilariasis [4–7], leishmaniasis [8–15] and trypanosomiasis [8, 16–23].

The data regarding vector-borne bacterial diseases and their prevalence in dogs are scarce. *Anaplasma platys*, has been detected in dog blood with a prevalence ranging between 18.6% in sick animals [2] and 13.5% in feral dogs [24, 25]. *Ehrlichia canis* has been recorded with a prevalence of 7% in sick dogs [2]; interestingly, a recent study performed at a clinic in Rosario, Santa Fé, showed that 76 of 100 dogs with pruritus presented structures compatible with hemotropic mycoplasmas, as determined by blood smears, and 91% of them presented varying degrees of anemia, as determined by hematocrit values and erythrocyte concentration [26]. No record of prevalence data is available for *Rickettsia* spp. or *Bartonella* spp. in Argentinian dogs.

The objective of this survey was to investigate the frequency and distribution of selected tick-borne pathogens in dogs from five locations in two provinces of Argentina. Microscopic blood smear examination as well as polymerase chain reaction (PCR) amplification using specific markers for the genera *Anaplasma*, *Babesia*, *Bartonella*, *Ehrlichia*, *Mycoplasma* and *Rickettsia*, followed by DNA sequencing were used to establish the prevalence of each infecting pathogen.

## Methods

### Animals, study site, and sample collection

In this study, a convenience sample of 70 dogs was tested from five locations: Isla Verde in Córdoba, (*n* = 24), San Lorenzo, Santa Fé (*n* = 24); Chañar Ladeado, Santa Fé (*n* = 2); Roldán in Santa Fé (*n* = 20); and Villa Constitución, Santa Fé (*n* = 4). Blood samples (3 ml) were collected by venipuncture in EDTA tubes. The population consisted of male (*n* = 26) and female (*n* = 44) dogs of different breeds and various ages, ranging from 1 to 10 years. Based on behavior and gross body examination, all animals appeared healthy. No detailed clinical examinations were conducted.

### Microscopic examination

Immediately after sample collection, peripheral blood smears were evaluated microscopically using May-Grünwald Giemsa method, for the presence of piroplasms (*Babesia* spp. or *Theileria* spp.), inclusion bodies (*Anaplasma* spp. or *Ehrlichia* spp.), or small basophilic structures on erythrocytes as indication of hemotropic mycoplasma infections.

### DNA isolation and PCR amplification

Genomic DNA extraction was performed using the QIAamp DNA Blood Mini kits (Qiagen, Valencia, USA) according to the manufacturer’s protocol. Eight microbial genera (*Anaplasma*, *Babesia*, *Bartonella*, *Borrelia*, *Ehrlichia*, *Francisella*, *Mycoplasma* and *Rickettsia*) were targeted using specific PCR assays as previously described [27].

## Results

### Blood smears

Peripheral blood smear analysis showed 81% (57/70) prevalence of structures morphologically compatible with hemotropic mycoplasmas when blood was evaluated microscopically using May-Grünwald Giemsa method. No structures resembling either piroplasms or *Anaplasma*/*Ehrlichia* were detected.

### Amplification and identification of vector-borne pathogens

A total of 55 of the 70 blood DNA tested (78.6%) were positive for at least one canine vector-borne pathogen (see Table 1).

**Table 1:** Prevalence (infected/examined) of different vector-borne pathogens in dog blood samples from Santa Fé and Córdoba, Argentina

Location	*Anaplasma* spp.	*Babesia* spp.	*Bartonella* spp.	*Mycoplasma* spp.
Isla Verde (Córdoba)	3/24	2/21	1/22	20/24
San Lorenzo (Santa Fé)	8/20	2/20	0/20	18/20
Roldán (Santa Fé)	0/20	1/18	1/18	13/20
Chañar Ladeado (Santa Fé)	0/2	0/2	0/2	1/2
Villa Constitución (Santa Fé)	0/4	0/4	0/4	2/4
Overall prevalence (%)	11/70 (15.7%)	5/65 (7.7%)	2/66 (3%)	54/70 (77.1%)

### Sequencing

In all cases in which blood samples were PCR positive, direct DNA sequencing was performed to identify pathogen species down to the level of strain when possible. Reference sequences for this study included the following GenBank accession numbers: AY055469 (*Anaplasma phagocytophilum*); AF399916 (*Anaplasma platys*); KU710803 (*Babesia vogeli*); AY618928 (large unnamed *Babesia* sp. “coco”); AF271081 (*Babesia gibsoni*); NC_005956.1 (*Bartonella henselae* Houston I); AF369529 (*B. henselae* SA2); DQ059763 (*Bartonella vinsonii* *berkhoffii* genotype II); DQ059764 (*B. vinsonii* *berkhoffii* genotype III); AF312490 (*Bartonella koehlerae*); AB896695 (*Bartonella clarridgeia*e); NC_007354 (*Ehrlichia canis*); NR_044747 (*Ehrlichia ewingii*); AY529641 (*Mycoplasma haemocanis*); GQ129113 (*Mycoplasma haematoparvum*); NR_103930 and AB610849 (*Mycoplasma suis*); and CP000848 (*Rickettsia rickettsii*). Sequences were compared to the GenBank database using the Basic Local Alignment Search Tool and homology data was generated using AlignX (Vector NTI suite 11.5.1, Invitrogen Inc., USA) software.

Hemotropic *Mycoplasma* spp. were the most prevalent pathogens detected and identified by DNA sequencing with an overall prevalence of 77.1%. *Anaplasma* spp. was detected and identified in 11 of the 70 (15.7%) dogs, meanwhile *Bartonella* spp. and *Babesia* spp. were detected in two dogs each.

The species identified at each genus and the species-specific prevalence are detailed in Table 2. Briefly, *M. haematoparvum* (identical with *M. haematoparvum* GQ129113; 544/544 bp) was detected at 31.4% prevalence; *M. haemocanis* (identical with *M. haemocanis* AY529641; 537/537 bp) at 48.6%; *M. suis* (identical with *M. suis* AB610849; 535/535 bp) at 2.9%; *B. clarridgeiae* (identical with *B. clarridgei*ae AB896695; 505/505 bp) at 1.5%; an uncharacterized *Bartonella* sp. (with a 552/582 bp homology with *Bartonella tribocorum* AF312505) at 1.5%; *B. vogeli* (identical with *B. vogeli* KU710803; 605/605 bp) at a prevalence of 7.7%, and *A. platys* (with a 77/79 bp homology to *A. platys* AF399916) at 15.7% prevalence.

**Table 2:** Prevalence (infected/examined) of *Mycoplasma* (hemotropic group) spp. in dog blood samples from Santa Fé and Córdoba, Argentina

Location	*M. haemocanis*	*M. haematoparvum*	*M. suis*
Isla Verde (Córdoba)	16/24	4/24	0/24
San Lorenzo (Santa Fé)	9/20	9/20	0/20
Roldán (Santa Fé)	7/20	8/20	2/20
Chañar Ladeado (Santa Fé)	0/2	1/2	0/2
Villa Constitución (Santa Fé)	2/4	0/4	0/4

Co-infections with two or more pathogens were also identified: *M. haemocanis* and *M. haematoparvum* were detected in 5.7% of the dogs (all from Roldán, Santa Fé); *M. haemocanis* and *A. platys* in five dogs (7.1%), *M. haematoparvum* and *A. platys* in six dogs (8.6%), and *M. haemocanis* and *B. vogeli* in two dogs (3%). All *A. platys*-infected dogs were also infected with a *Mycoplasma* spp. (either *M. haemocanis* or *M. haematoparvum*).

No amplification of *Borrelia* spp., *Ehrlichia* spp., *Francisella* spp. or *Rickettsia* spp. DNA was obtained in any of the 70 samples tested.

## Discussion

The detection of canine vector-borne pathogens represents a constant challenge for veterinarians and researchers. The wide array of vector-borne pathogens (protozoans, bacteria and viruses), their diverse biological behavior and distribution, and the wide spectrum of clinical signs contribute to the difficulties associated with the diagnosis of CVBD [28, 29]. In Argentina, there are only a few reports available for canine tick-borne diseases caused by species of the genera *Anaplasma*, *Babesia* and *Ehrlichia*, and most of them have been limited to the state of Buenos Aires. *Babesia vogeli*, one of the most common etiological agents of canine babesiosis in South America, was recorded based on molecular characterisation for the first time in two dogs from Buenos Aires in 2008 [1]; *A. platys*, the etiological agent of granulocytic anaplasmosis and cyclic thrombocytopenia, was detected in sick and feral dogs, and *E. canis*, the agent of canine monocytic erhlichiosis, was also detected in feral dogs [2]. To date, no records on the detection or prevalence of *Rickettsia* spp., *Bartonella* spp. or *Mycoplasma* (hemotropic group) spp. are available from dogs in Argentina. The objective of this survey was to investigate the prevalence of tick-borne pathogens in dogs in five locations from two states of Argentina (Santa Fé and Córdoba). PCR amplification, aimed at genera *Anaplasma*, *Babesia*, *Bartonella*, *Borrelia*, *Ehrlichia*, *Francisella*, *Mycoplasma* and *Rickettsia*, followed by DNA sequencing was used to establish the presence of each infecting pathogen in blood collected from 70 dogs.

The prevalence of *Anaplasma plat*ys found in dogs from Córdoba (12%) and Santa Fé (17.8%) was similar to that previously reported for dogs from Buenos Aires (13.5–8.6%) [2, 24, 25]. A higher prevalence of *Babesia vogeli* was detected in Córdoba (10%) and Santa Fé (6.8%) compared with a previously reported prevalence of 0.2% from Buenos Aires [1], even though the latter was estimated using blood smear analysis. No amplification of *Borrelia* spp., *Ehrlichia* spp., *Francisella* spp., or *Rickettsia* spp. DNA was obtained in any of the samples tested in the present study.

Interestingly, this is the first report on the detection and molecular identification of several *Mycoplasma* species from dogs from Argentina: *M. haemocanis*, detected in 16/24 (66.7%) and 18/46 (39.1%) of dogs from Córdoba and Santa Fé, respectively; *M. haematoparvum*, with a prevalence of 16.7 and 39.1% from Córdoba and Santa Fé, respectively, and *Mycoplasma suis* detected in two dogs from Roldán. More importantly, this is the first report of the detection and amplification of *Mycoplasma suis* DNA from dog blood.

No statistically significant association was found (*P*-value of 0.7226, using a 2×2 contingency table Fisher’s exact test) between blood smear results and DNA amplification, demonstrating that microscopic analysis of blood smear is very unspecific with very low sensitivity when compared with PCR.

Several previously unrecognized or neglected vector-borne pathogens that affect companion animals are present in Latin America, and specifically, Argentina. The data presented here show that zoonotic pathogens of the genera *Anaplasma*, *Babesia*, *Bartonella* and *Mycoplasma* occur in variable prevalences in dogs without any visible sign of infection or clinical signs.

Unfortunately, vector-borne diseases are among the most complex of all infectious diseases to diagnose, mitigate, control and prevent. In this work, we presented evidence of several previously unreported infections in dogs from Argentina: *Bartonella clarridgeiae*, an uncharacterized *Bartonella* sp. (closely related to *B. tribocorum*), and three hemotropic *Mycoplasma* spp. (*M. haematoparvum*, *M. haemocanis* and *M. suis*).

## Conclusions

Most if not all of the bacterial pathogens detected during this study are zoonotic [30–33], which not only represent risk for companion animals, but also for people. Unfortunately, the information about the importance and risks related to canine vector-borne pathogens available to veterinarians, public, and the medical community, is very scarce, limited, or simply inexistent. In that sense, to address the challenges that CVBD impose to the region, significant improvements in clinical diagnosis, medical practices, and vector control and surveillance, should be implemented.

## Abbreviations

CVBD: Canine vector-borne diseases
EDTA: Ethylenediaminetetraacetic acid
PCR: Polymerase chain reaction
